# “I did not know about all these”: Perceptions regarding safer conception methods by women living with HIV in Gaborone, Botswana

**DOI:** 10.1371/journal.pone.0242992

**Published:** 2020-12-01

**Authors:** Sarah A. Gutin, Gary W. Harper, Neo Moshashane, Kehumile Ramontshonyana, Atlang Mompe, Paul J. Fleming, Jane Harries, Doreen Ramogola-Masire, Chelsea Morroni

**Affiliations:** 1 Dept. of Health Behavior and Health Education, School of Public Health, University of Michigan, Ann Arbor, Michigan, United States of America; 2 Women’s Health Research Unit, School of Public Health and Family Medicine, Faculty of Health Sciences, University of Cape Town, Cape Town, South Africa; 3 Division of Prevention Science, Center for AIDS Prevention Studies, University of California, San Francisco, San Francisco, California, United States of America; 4 Botswana—University of Pennsylvania Partnership, University of Botswana Main Campus, Gaborone, Botswana; 5 Department of Obstetrics and Gynaecology, Faculty of Medicine, University of Botswana, Gaborone, Botswana; 6 Liverpool School of Tropical Medicine, Liverpool, United Kingdom; University of North Carolina at Chapel Hill, UNITED STATES

## Abstract

Various safer conception methods to limit HIV transmission risks can be offered in resource-constrained settings. However, implementation of safer conception services remains limited in many countries, including Botswana. Understanding perceptions about safer conception methods and the benefits and challenges to use can help with the development of policies, interventions, and service delivery models. Forty-five women living with HIV in the greater Gaborone, Botswana area participated in focus group discussions. Themes were analyzed using interpretive phenomenology. Despite low knowledge of specific safer conception methods that can be used to prevent transmission of HIV when trying to achieve pregnancy, there was noted interest in pre-exposure prophylaxis and vaginal insemination. Challenges to greater uptake were noted including a lack of knowledge about a range of SC methods, limited partner support and communication, provider stigma, health systems barriers, current policies, and the cultural acceptability of methods. Interventions will need to address these challenges and be responsive to the needs and reflect the realities of WLHIV who desire pregnancy in order for safer conception uptake to become a common practice.

## Introduction

The majority of the 36.9 million people living with HIV (PLHIV) worldwide are in their reproductive years [1]. Data from various sub-Saharan African countries suggests that childbearing desires among men and women living with HIV remain strong with 20 to 51% expressing a desire for children [2–5]. Many new HIV infections in sub-Saharan Africa occur in long-term sero-different relationships [6, 7], and may be due to attempts to become pregnant [8–10]. In this context, acceptable and effective safer conception (SC) techniques that can offer opportunities to reduce the risk of horizontal transmission to partners are especially important to stemming HIV transmissions. Also, while SC is most applicable for sero-different couples, there are also SC benefits for sero-concordant couples such as preventing HIV superinfection and transmission of drug resistant virus [11].

SC methods for resource-limited settings include the use of behavioral and pharmacologic reproductive strategies. Some methods, such as condomless sex limited to the time of peak fertility whether the male partner is living with HIV or not (known as timed condomless intercourse) [12] and vaginal self-insemination when the female partner is living with HIV and the male partner is not living with HIV, are conception specific [11, 13]. Other strategies, such as antiretroviral therapy (ART) to suppress the viral load of the PLHIV [14, 15], the use of oral pre-exposure prophylaxis (PrEP) by the partner who does not have HIV [16–18], and medical male circumcision for HIV uninfected male partners [19, 20] are not conception specific but are valuable SC options. Studies in Kenya, Uganda, and South Africa have indicated that all of these methods are acceptable and have been used by HIV-affected couples [21–23]. Better understanding what SC methods people are knowledgeable of and which methods are acceptable and feasible within relationships could help increase SC uptake in sub-Saharan Africa.

The implementation of SC services for HIV-affected couples within healthcare settings remains scant in many resource-limited countries, including Botswana. In Botswana, 22.2–27.3% of women 15–49 years are living with HIV [24] and between 60–70% of women know they are living with HIV before becoming pregnant [25, 26]. While Botswana is expanding ART coverage and has signed on to the UNAIDS 95-95-95 targets (95% HIV counseling and testing, 95% ART initiation, 95% viral suppression), the HIV incidence rate in the country indicates substantial ongoing transmissions [27]. This highlights the need for SC methods that can complement ART in reducing horizontal transmissions. While the most recent Botswana HIV clinical care guidelines mention safer conception strategies, little guidance has been offered on how to counsel couples on using specific approaches. SC services have not yet been made routinely available in public sector clinics, some methods (such as PrEP) are only available in the private sector or through demonstration projects [28], and providers have not been formally trained on these methods.

Few inquiries in high HIV prevalence settings have compared the perceptions of PLHIV across SC strategies [29]. In this study, we sought to gain an in-depth understanding of the perceptions of women living with HIV (WLHIV) in Botswana regarding different SC methods, including their perceived benefits and challenges. We explored knowledge and perceptions toward specific SC methods that could be made available in public sector clinics. Understanding the perspectives of WLHIV and the cultural context in which techniques would be used is important to developing relevant interventions. Insights from this study will inform policies and interventions that support HIV-affected couples to safely achieve their fertility goals by addressing challenges and offering a range of SC options that best meet their needs.

## Methods

### Study design and population

Informed by the information, motivation, and behavioral skills (IMB) model [30], eight qualitative elicitation focus group discussions (FGDs) took place between February and June 2018 in the greater Gaborone, Botswana area. A group setting was ideal for investigating these phenomena because the interaction between participants allows members to hear, reflect, and then elaborate on the comments of others. In this way, participants were able to agree or disagree, react to various opinions, and explain their views when encouraged or challenged by other participants [31]. This approach helped to validate the points being raised as shared experiences.

All WLHIV were recruited for FGDs from Botswana Network of People Living with HIV (BONEPWA+) affiliated support organizations. BONEPWA+ is a national umbrella body formed in 2000 by and for PLHIV. BONEPWA+ coordinates and manages support groups for PLHIV, provides empowering skills and strategies, and helps strengthen linkages between prevention, care, and support services. WLHIV were sampled based on age (18 to 60 years) and pregnancy history (never pregnant, currently pregnant, not currently pregnant but already mothers) so a range of possible experiences and attitudes would emerge. Partner sero-status was not an inclusion criteria as SC can benefit both sero-different and sero-concordant couples. In addition, a stated desire for future pregnancies was also not an inclusion criteria as women can accurately share their reflections about SC methods regardless of fertility intentions. We had planned that the upper limit of our age range would be 45 years but due to community requests, and a desire to be responsive and respectful, we extended our age range to 60 years. By using this sampling technique, the intent was not to create a representative sample, but rather, to capture various viewpoints and the lived experiences of WLHIV, who primarily access SC services [32].

WLHIV were recruited through support organizations using one of two approaches. First, community-based research advocates recruited participants following support group meetings. Second, posted flyers at support group sites contained basic information about the study and contact information so interested women could contact the PI. Study coordinators screened WLHIV and explained study aims. WLHIV were reimbursed 30 Botswana Pula (approximately $3 USD) to cover local transport costs and were offered snacks during FGDs.

Ethical approvals were obtained from the University of Michigan Health Sciences and Behavioral Sciences Institutional Review Board (Ann Arbor, Michigan), the University of Botswana Research Ethics Committee, Office of Research and Development (Gaborone, Botswana), and the Health Research and Development Division of the Botswana Ministry of Health. Permission was also obtained from the Executive Director of BONEPWA+ and support group leaders before recruitment of WLHIV took place. All participants were provided with a written statement regarding the research and their rights before providing written informed consent. In addition, all participants signed a confidentiality agreement that stipulated that all FGD members understood that what was discussed in the group was private and confidential.

### Data collection and analysis

FGDs explored the perceptions of WLHIV in Botswana regarding different SC methods, including their perceived benefits and challenges. The data collection and analytic process were informed by a social constructivist framework [33] through a desire to capture and report multiple experiences and perspectives so as to develop a deeper understanding of a particular context. This allowed women to define and describe the concepts and ideas related to SC in their own words and to articulate how SC is understood and practiced (or intended to be practiced) within their community and cultural context.

The interview guide was drafted, tested, and revised through a collaborative process involving the PI (an English-speaking, non-African White female, sexual and reproductive health (SRH) researcher from the USA who is a mother and is not living with HIV), two experts in the field of SRH/SC (one doctor from Botswana, one researcher from the USA), two local researchers with many years of experience in SRH/HIV research in Botswana, and advocates for PLHIV, to ensure exploration of appropriate constructs. All members of the study team conduct behavioral research focused on SRH amongst PLHIV. FGD domains included information about SC methods (e.g. methods with which WLHIV were familiar, methods for which they would like more information) and perspectives (SC methods in which they were most interested, methods in which they were least interested, perceived benefits and challenges to use).

Local, female research assistants conducted FGDs in Setswana (the local language) and English. Research assistants were experienced qualitative interviewers with expertise in HIV/SRH. All FGDs took place in private rooms at the BONEPWA+ offices in Gaborone, in the location where the support group met, or at the University of Botswana main campus. FGDs consisted of 3 to 9 WLHIV per group. Discussions lasted one hour and 42 minutes on average (ranging from one hour to two hours and 17 minutes). Interactive techniques including a small group breakout session, the use of cards with SC methods and information on them, and pile sorting were used to enhance comfort, participation, and to gain depth in understanding.

To assess the perspectives of WLHIV about SC methods, each participant received a set of five cards that described basic information about various SC methods, including PrEP, vaginal insemination, timed condomless intercourse (referred to as timed unprotected intercourse at the time of the FGDs), medical male circumcision, and ART (see Table 1 for more detail, full cards available as a supplement). After reviewing each card, participants formed small groups to discuss the methods further. These small groups allowed for greater participation and increased comfort when discussing this intimate topic. After discussing methods in small groups, women were asked to put the cards in a pile based on which method they would be most interested in using (with the one they were most interested in on top, and the one they were least interested in on the bottom). Pile-sorting data is only available for 34 of the 45 FGD members. The full group of women were then asked to reflect on why they were most interested in their top choice method and why they were least interested in their last choice method. They were also asked about which methods they thought their partners would consider using and which methods they desired more information about. FGDs were transcribed verbatim from digital recordings in the language the interview was conducted and then translated into English. A member of the study team reviewed each transcript for quality and accuracy and corrections were made when necessary.

**Table 1:** Information about safer conception methods on cards shared during FGDs.

Method	Information listed about method
PrEP	• Treating the HIV-negative partner with ARV pills called pre-exposure prophylaxis, or PrEP, during the time they are trying to get pregnant • Couple has sex without a condom during the days during a woman’s cycle when she is most likely to get pregnant
Vaginal insemination	• Semen from an HIV-negative man is collected in a condom or cup • Then, in a syringe, the semen is taken from the condom/cup and gently placed inside the woman • Usually done at home.
Timed unprotected intercourse	• Every month during a woman’s cycle, there are a few days when she is most fertile (most likely get pregnant). • A woman can track her cycle to figure out the days when she is most likely to get pregnant. • A couple then has sex without a condom during those days to try and increase the chance of getting pregnant.
Medical male circumcision	• HIV-negative men have a surgical procedure where the foreskin of the penis is removed. This can reduce the chance of the man getting HIV during unprotected sex. • Men choosing this method will need to wait at least 6 weeks after VMMC before having sex.
ART	• Treating the HIV-positive partner with ARVs • Checking their HIV viral load to make sure it is very low or ‘undetectable’ • Then the couple has sex without a condom during the days during the woman’s cycle when she is most likely to get pregnant

The data for this study were derived from eight FGDs with a total of 45 WLHIV. Data analysis was guided by an interpretive phenomenological approach [34]. Interpretive phenomenology focuses on understanding people’s perceptions, perspectives, and lived experiences by prioritizing the participant viewpoint. This also allows one to examine the social/cultural contexts in which the data emerged. This is a useful approach because it can be used to examine the rationale and motivations for engaging in a behavior or not, and it is a research method that is capable of examining the role of social norms in individual’s lives [35]. The method has also been used to understand how people are affected when their sexual behaviors are defined as problematic and are stigmatized [35]. This makes interpretive phenomenology useful for examining SC amongst WLHIV because childbearing amongst PLHIV has been stigmatized.

All FGDs were coded by two independent coders (the PI and one local research assistant) to ensure reliability. Data analysis began by reading hard copy transcripts, creating memos, and assigning initial codes [36]. Using this process, we identified recurring themes and developed descriptive codes to complement initial deductive codes, which were derived from the research questions [36]. Following initial analysis and codebook finalization, translated interviews were entered into the web application Dedoose (www.dedoose.com) for final code application and assistance in systematic data management [37]. We reviewed coded text thematically and then conducted cross-case analysis to deepen our understanding by examining similarities and differences and to better understand recurring themes [36]. Initial findings were reviewed by returning to the relevant segments in each transcript to ensure that the context and meaning were preserved. In cases where there was disagreement about interpretation, team members discussed discrepancies until consensus was achieved. In the section that follows, we describe the concepts and themes that emerged using illustrative quotes as examples.

## Results

Participants were 19 to 60 years old, with a mean age of 37 years (nine women were over 45). Most participants (93%) had ever been pregnant and four women were pregnant at the time of the FGDs. Fourteen women had learned their HIV status during a pregnancy and 20 women had become pregnant since learning their HIV status. In addition, 93% had at least one child with a mean of 2.4 children per woman (range 0–7). Some women specifically mentioned during the interview that they would like to have another child but we did not ask a specific question about their personal fertility intentions. On average, women had been diagnosed with HIV 10 years ago (in 2008, range 1993–2018). ART status was not an eligibility criteria for the study, but 33 women said during the interviews that they were using ART. Similarly, not all women on ART shared how long they had been on treatment. However, of those who shared this information, they had been on ART for an average of 10 years (2008, range 2001–2015). In addition, partner sero-status was not an inclusion criteria and women were not specifically asked about the HIV status of their partners. However, some women chose to share this information during the FGDs and participants reported that they were in both sero-concordant and sero-different relationships. We noted no major differences in responses across these socio-demographic categories and so we present our findings together.

The results are organized to examine themes under two primary categories. First we discuss information, perceptions, benefits, and challenges for each SC method discussed. Next, we discuss benefits and challenges that cut across all methods and could aid or hinder greater SC method uptake.

### Information, perceptions, benefits, and challenges by method

During FGDs, WLHIV were presented with and discussed five SC methods: PrEP, vaginal insemination, timed condomless intercourse, medical male circumcision, and ART. Women mentioned the perceived benefits and challenges of PrEP and vaginal insemination in greater depth than those for timed condomless intercourse, medical male circumcision, or ART even though PrEP and vaginal insemination were approaches that were less well known (see Table 2).

**Table 2:** Perceived benefits and challenges by safer conception method as reported by WLHIV in FGDs.

Method	Benefits	Challenges
PrEP	1. Preserves intimacy/physical connection 2. Makes conception feel more natural 3. Added transmission protection for partner 4. Easy to use 5. Forces couple communication	1. Availability–only in private sector 2. Cost–too expensive 3. Access–where to get PrEP 4. Ensuring partner adherence
Vaginal insemination	1. No HIV transmission risk 2. Inexpensive	1. Method not culturally acceptable 2. Unnatural/ reduces intimacy 3. Concerns about using the technique at home without healthcare provider 4. Associations with cattle breeding
Timed condomless intercourse		1. HIV transmission risks for partner 2. Risks for other sexually transmitted infections
Medical male circumcision	1. Accessible across Botswana 2. Well known method 3. Reduces chances of HIV transmission to partner	1. Male resistance/ refuse method 2. Rumors 3. Unnatural 4. Only for men not living with HIV
ART	1. Method is well known 2. Viral suppression reduces transmission to one’s partner	1. Inconsistent ART adherence 2. Rumors

#### PrEP information and perceptions

Despite not having heard much about PrEP, after reading a short description of the method, WLHIV had an overwhelmingly positive response to the use of PrEP by their male partners who are not living with HIV as a SC method. PrEP was the SC method that the majority of women said they were most interested in using (18/34 women) and many wanted more information about the method.

#### PrEP benefits

The benefits of PrEP discussed by women centered on five main areas: preserving sexual intimacy, feeling natural, offering additional HIV transmission protection, ease of use, and necessitating couple communication. WLHIV liked PrEP because they felt it preserved sexual intimacy/physical connection and made the conception process feel more natural as compared to some other SC options. A key benefit was that PrEP offered added protection for their partners above their personal viral suppression when they were trying to become pregnant. Women also valued that PrEP is an easy to use method that forces couples to communicate and discuss pregnancy plans so they are in agreement before trying to have children.

I feel that it’s the best because there is intimacy and connection … I mean there is bonding as compared to the artificial insemination one [referring to vaginal insemination]. (26 years)

Some [partners] wouldn’t want to just say ok we can have unprotected sex. They still have that thought at the back of their minds “ok what if she infects me?” So like this thing [PrEP] it will only be an assurance to them that you are fully protected now. (35 years)

There is an agreement between two people that they want to have a child. Like right now, I am pregnant and that was not my intention to, we didn’t agree on the pregnancy so with this method there is no such thing, there has to be an agreement. (29 years)

#### PrEP challenges

Although women were interested in PrEP as a SC option, they also mentioned concerns focused around four areas: Availability of PrEP, cost, knowing where to go for services, and concerns about proper PrEP adherence by partners. WLHIV were concerned that PrEP was only available in the private sector and therefore out of reach for most people in Botswana, who predominantly access care in the public sector where services are low cost or free. Since PrEP has generally been made available in the private sector so far, the costs associated with its use make the method too expensive for many couples who normally access free or heavily subsidized care in the public sector. Since PrEP is also a fairly new method in Botswana, women were not clear about where to seek PrEP for SC. Lastly, some women had concerns about partner adherence to PrEP and making sure partners took the pills correctly so they would receive the added protection that PrEP could offer.

We could say PrEP … can be accessed by people who perhaps have medical assistance in the form of medical aid [insurance] and they are working but an ordinary Motswana is not privileged enough to get this service. (34 years)

When you go to your Doctor and they write that prescription … it’s mostly expensive because now I’ll take that prescription, go to the pharmacy and buy that bottle of about P500 [500 pula, approximately $50 USD]. That’s how it is. (35 years)

I do not trust this pill method [PrEP]. A person will be given these to take at home right? I will not be sure that they take them properly and in time. (29 years)

#### Vaginal insemination information and perceptions

Although vaginal insemination was not a SC method that many WLHIV were aware of, some women had a positive response to the method. Many women (14/34) said that vaginal insemination was the SC method they would be most interested in using with their male partners who are not living with HIV. Vaginal insemination was the method that women most wanted more information about.

#### Vaginal insemination benefits

The benefits of the method focused around two main areas: eliminating HIV transmission risk when trying to conceive and the low cost of the method. The most commonly mentioned reason for why women liked vaginal insemination was that it was a method that ensured negative partners would not be at any risk for HIV transmission. In addition, it was mentioned that vaginal insemination is an inexpensive SC option.

I like vaginal insemination because you will be 100% sure that there is no contact of the virus. (37 years)

I didn’t know that it was the easiest and most cheapest method … I even went to buy the syringe … and it was about P2 [2 pula, approximately 20 US cents]. So … this is the cheapest. So I like it for that. (35 years)

#### Vaginal insemination challenges

Vaginal insemination was not a well-known method and women mentioned challenges that were method-specific. Challenges focused around three areas: the cultural acceptability of the method, a sense that the method was unnatural and reduced intimacy between partners, and concerns about using the technique at home without the assistance of a healthcare provider. A commonly mentioned barrier was that vaginal insemination would not be culturally acceptable and as such, men might be distrustful of this method and doubt the paternity of babies born using this technique. There was also some concern that men’s masculinity might be in question using an insemination method, further making the method less culturally acceptable. Women also felt this approach lacked intimacy and desired a method that promoted bonding with their partners when trying to conceive. Women also voiced concerns about the mechanics of using a vaginal insemination technique at home and mentioned a need for training before attempting to use the method at home, unassisted by a healthcare provider. Finally, Botswana is a country with a strong connection to cattle herding and animal husbandry. A common rumor was that insemination approaches were only used with cattle.

I would be more interested in vaginal insemination… but I am sure my partner would not be interested in it. Batswana men believe in a normal and straightforward sexual intercourse, skin to skin. (37 years)

I feel that … after falling pregnant … they [your partner] may backtrack or change their mind and blame the pregnancy on you, saying since its artificial insemination you are responsible for making yourself pregnant. They will [say] you injected yourself in order to be pregnant and they had nothing to do with the pregnancy. And also there is no intimacy, it’s like science stuff. (29 years)

Vaginal insemination, how sure will the healthcare workers be that you followed the correct procedures at home? (32 years)

#### Timed condomless intercourse information, perceptions, and challenges

Many women had heard of timed condomless intercourse as a SC technique and knew that it should be used in conjunction with another method, such as viral suppression, in order to protect partners when having condomless sex during a woman’s fertile days. However, some women were least interested in using timed condomless intercourse and distrustful of the method because of the perceived risks for their partner and personal risks for acquiring other diseases/sexually transmitted infections.

I’ve heard that if my boyfriend is HIV negative … we can go to the hospital and then they can introduce him into this medication [PrEP for the male partner] and then monitor my cycle to see when I can fall pregnant. And then that is the time that we can have unprotected sex. (29 years)

The timed unprotected intercourse has more risks … in terms of infection and reinfection. And it’s not really, really accurate. … It’s increasing the risk of infection. (34 years)

#### Medical male circumcision information, perceptions, benefits and challenges

Many women had heard of medical male circumcision as a SC technique that could be used by men who are not living with HIV. However, despite some benefits, many challenges were discussed. Noted benefits were that medical male circumcision was a well-known option and was accessible across Botswana. Challenges focused around four main areas: male resistance to the method, rumors, a feeling that the method was unnatural, and that it had limited use because it could only be used by men who were not living with HIV. Women noted that men were resistant to or refused medical male circumcision as an HIV prevention approach. Women said that medical male circumcision was rumored to lower sexual desire, arousal, and performance and that some men felt the practice was unnatural. Some women also felt the option was not ideal because it could only be used as an HIV prevention technique by men who were not living with HIV.

Most males they are difficult when it comes to safe male circumcision. They refuse. … yes it reduces chances of getting infected but most guys don’t circumcise. (21 years)

I once met someone who said that circumcision reduces the degree of a man’s sensitivity. (37 years)

#### ART information, perceptions, benefits, and challenges

The use of ART to achieve viral suppression and the subsequent benefits it could offer for SC were well understood. However, women seemed least interested in using ART as a SC technique–mostly because it was a method that was already well known. Although they mentioned positive outcomes as a result of ART adherence, women did not explicitly discuss benefits of ART. The main challenge that women noted focused on treatment adherence. WLHIV reported that although ART was a well-known method, it was not always used consistently.

We have been told that the Treat all program is the best, because you start treatment with viral suppression, which means the virus will always be undetectable or low, then the chances that you can pass the virus on to your partner are very low, even when you are pregnant the chances of passing the virus on to the baby during birth are low. (48 years)

Mostly they [WLHIV] know this one [ART] …But even though they know it, it’s not like they use it. (39 years)

### General influences on SC method use

WLHIV described a number of crosscutting issues that affect SC method uptake. We first describe facilitators to SC method uptake followed by a discussion of noted challenges. The involvement and participation of partners was a theme that came up as a facilitator and a possible barrier, and these will be discussed separately.

#### General SC facilitators

Women discussed facilitators to the use of SC methods and many of these highlight the important role of partners in SC decision-making and use. Important facilitators included the desire to have children, protecting partners from HIV transmission, male partner communication and support, and partner agreement with SC method choices. For example, WLHIV are motivated by the ability to protect their partner from HIV infection when their partner is not living with HIV. In addition, partner disclosure and partner communication were very important. Women also highlighted the importance of choosing a SC method that their partner would agree to use. ART, PrEP, and vaginal insemination were mentioned as methods that partners might consider trying.

When it comes to me being positive and my partner being negative, I have to be the one to protect him from being infected. … I’d be so interested in using any method that we both agree on … It’s very, very important so that he won’t get infected. (35 years)

My partner wants a baby but I have been delaying it because I was afraid of my HIV status but since I know these methods now I know there are chances of having a baby and I can use these. (21 years)

#### General SC challenges

Women also noted important challenges to greater SC uptake. Themes included a lack of counseling and information about SC methods, not knowing where to seek services, concerns about stigma from providers, and unsupportive male partners. A key challenge was the lack of counseling about SC and a sense that SC methods are not well known or understood by WLHIV. As a result, women also did not know whom to go to when they were in need of SC services. Some women also said that internalized stigma and anticipated stigma and discrimination from providers hinders women from seeking SC services. Women felt that internalized stigma and shame led some women not to disclose their HIV status. In addition, some women anticipated stigma and feared negative responses from providers when they wanted to have children. Women made a connection between overworked healthcare workers and long queues and the effect that these structural barriers can have on perpetuating stigma. In addition, while partner communication and agreement to use SC methods was mentioned as an important facilitator, a lack of partner support was seen as a key challenge to greater SC uptake. Women mentioned a number of challenges to SC posed by male partners including a refusal to use SC methods that they felt were unnatural, a desire by men to have condomless sex when trying to get pregnant (as opposed to using an assisted reproductive technology), and men not wanting to attend clinic visits where they could be counseled about SC.

We are never told about these methods. We just find ourselves pregnant that’s when we are told that we people living with HIV are not supposed to fall pregnant many times. Honestly we are never taught about these methods. (37 years)

Sometimes they [nurses] can say offensive things to you because they are swamped with work and long queues outside, and you will wonder how a person can say such hurtful words to you. (age unreported)

Tswana men will not [agree to use SC methods]. As a Setswana woman … all you want is a child. … Then you say let us do this to protect our baby, protect you as well my man. He will refuse.” (49 years)

## Discussion

PLHIV must be assisted to make conception as safe as possible. SC methods can be made available to HIV-affected couples in resource-constrained settings. However, to offer SC services in Botswana and similar contexts, it is imperative to understand perceptions about different methods and benefits and challenges to use. In this study, where we asked WLHIV to compare SC methods, interest in these strategies was high although there was a lack of prior knowledge and counseling about SC methods. WLHIV were most interested in and wanted more information about PrEP and vaginal insemination. Challenges to greater uptake were noted including a lack of knowledge about a range of SC methods, limited partner support and communication, provider stigma, cost and access for some methods, health systems barriers, current policies, and the cultural acceptability of methods. With this information, it is possible to develop interventions, service delivery models, and policies that are responsive to the needs and reflect the realities of WLHIV who desire pregnancy.

### General considerations for SC rollout in Botswana

#### Knowledge and counseling

Although a lack of prior knowledge and counseling about SC methods was mentioned as an important challenge to uptake, this did not seem to affect potential interest. This has also been seen in various sub-Saharan African settings where PLHIV have voiced a desire to speak with providers and learn more about SC, despite low knowledge about SC methods [38–40]. Although most women stated a preference for PrEP and vaginal insemination, it is clear that one size will not fit all couples. This data can help tailor educational campaigns and trainings so a range of SC methods are made routinely available to HIV-affected couples. In addition, demand creation campaigns that address individual level factors such as knowledge, motivation, and personal risks may help to increase uptake of methods. Demand creation campaigns could focus on educational materials for HIV-affected couples in order to address gaps in knowledge or on risk assessment tools that help couples to assess their personal risks for HIV transmission if they want to have children. By providing needed information, such materials can promote requests for SC services by clients.

#### Including partners

WLHIV highlighted the importance of fully involving male partners in SC method decision-making and use. Data from other sub-Saharan African countries has also highlighted the importance of partners in fertility-related desires and decisions, and around SC specifically, with male partner desires for children being especially dominant in relationships and contributing to HIV risk behaviors [2, 5, 8, 11, 22, 41, 42]. Typically, when they happen, fertility related discussions tend to focus on women because women most often seek care [43–45]. However, these discussions are also relevant for men, as men may be more, or just as likely, to desire childbearing [4].

In addition, most SC techniques require full partner participation and agreement for use. This highlights the important role of partner communication in both discussing pregnancy desires and then choosing a SC method, or mix of methods, that couples are comfortable with. In this study, ART, PrEP, and vaginal insemination were mentioned as methods that partners might agree to use. Fully involving partners in couples-based SC counseling and decision-making is imperative for the success of any SC program. However, including men in reproductive services may be challenging since health services often focus on the reproductive needs of women [46]. Men may be reluctant to come with their partners to healthcare services that are viewed as female environments [46]. Perhaps bearing this out, a SC implementation project in South Africa found that 55% of women were ever accompanied by their partners for SC services and 45% always attended alone [22]. Our results suggest that partner support and engagement and addressing concerns about masculinity will be critical to SC uptake in Botswana. Models that improve couples SC counseling and address cultural acceptability should see better outcomes. Although couples HIV counseling is limited and current country guidelines do not include couples counseling, couples SC interventions that are based outside of health centers, such as having community health worker visits in the home, may be more appealing to men and increase comfort and engagement.

#### Addressing stigma

Although Botswana has a long-standing HIV care program and has offered ART for over 15 years, women in this study described internalized stigma and anticipated stigma and discrimination from healthcare providers as challenges to greater SC uptake. Previous studies in Botswana have reported that stigma impacts HIV testing, disclosure, ART uptake, and adherence [47–51]. The desire for childbearing can create a conflict for WLHIV who wish to fulfill personal desires for children and cultural expectations of motherhood but also face strongly perceived community and provider disapproval associated with HIV and reproduction [40, 52–54]. In particular, the anticipation of stigma from providers may inhibit communication about fertility desires and SC. Although Ministry of Health and Wellness guidelines already support a reproductive rights approach to childbearing for PLHIV [55], encouraging providers to routinely assess fertility desires and counseling about family planning as well as SC may signal to PLHIV that childbearing is a topic that is not off limits.

#### Changing policy and guidelines

Women raised a number of issues that will require changes and guidance at the Ministry of Health policy level and that will impact the health systems level. Currently, formal SC services are not offered in public sector clinics in Botswana and government guidance on the appropriate package of services to offer PLHIV who desire to be pregnant has been limited [55]. As a first step, Ministry of Health and Wellness documents will need to be updated to provide more guidance about a range of possible SC approaches. Once guidelines have been updated, providers will need training so they can correctly counsel and offer SC services to HIV-affected couples in a supportive environment where reproductive rights are protected. In our work with providers in Botswana and in research in Uganda and South Africa, providers have voiced a desire for such trainings so they can better support PLHIV [40, 43, 56].

### Considerations by SC method

#### PrEP

Despite low knowledge about PrEP, WLHIV had a positive response to PrEP as a SC method. Women liked that PrEP preserved intimacy and offered added prevention benefits for partners. As has also been noted in Kenya, PrEP was viewed as a method that was culturally acceptable because it did not threaten masculinity and was seen as more “natural” [5]. However, women also noted potential barriers to use including access to PrEP, cost, knowing where to seek services, and concerns about proper PrEP adherence by partners. This concern about correct partner adherence has been noted elsewhere [57].

Although the most recent Botswana HIV clinical care guidelines suggest that PrEP could be appropriate for sero-different couples attempting to conceive, PrEP is not yet widely available in the public sector and little guidance has been offered on counseling couples on this strategy [55]. Although PrEP can be accessed in the private sector, the associated cost is prohibitive for many. In order to make PrEP a viable SC option in Botswana and expand access beyond the private sector, we recommend that the Ministry of Health and Wellness or donors make PrEP available free of charge in the public sector for sero-different partners. In addition, we recommend ongoing PrEP use in pregnancy among sero-different couples in which the woman is not living with HIV. Offering PrEP for free at public sector clinics across the country would likely address barriers related to access, availability, and cost and allow a wider segment of the population to benefit from this option. However, despite the perceived interest in PrEP for SC that has been noted here and elsewhere, interest and availability does not always translate into use [22, 57]. An expanded PrEP rollout for sero-different couples in Botswana would likely benefit from educational and demand creation campaigns.

#### Vaginal insemination

As has been noted in other contexts, some women were particularly interested in vaginal insemination techniques, expressed a preference for methods that did not involve direct sexual contact, and ensured that their negative partners would not be at risk for HIV transmission [22, 57]. This is not surprising as studies have found that PLHIV are concerned about transmitting HIV to their partners [5, 46]. However, as noted elsewhere, some important barriers to greater vaginal insemination uptake center around suspicion of artificial/assisted reproductive technologies, a feeling that insemination techniques are unnatural, the association between syringe insemination techniques and their use in cattle breeding, and concerns about cultural acceptability [5, 57, 58]. With proper education about vaginal insemination, it may be possible to dispel these concerns, especially since many women were interested in this technique despite limited knowledge of the method.

#### Timed condomless intercourse and medical male circumcision

Although both timed condomless intercourse and medical male circumcision were SC methods that women were aware of, as noted here and in South Africa [22], neither method was particularly popular. Also, as noted in South Africa, clients in this study were distrustful of condomless sex for fear of HIV transmission [22, 38]. Women in Botswana may need more counseling about the safety of combining timed condomless intercourse with viral suppression before they see timed condomless intercourse as an acceptable method.

#### Viral suppression with ART

Viral suppression with ART may be an important SC area to target. Women were aware of ART and its benefits for reducing partner transmission risks, and they understood the idea of U = U (Undetectable = Untransmittable or uninfectious), and the need to be virally suppressed before attempting pregnancy. However, women did not seem motivated to use ART as a SC technique, perhaps exhibiting treatment fatigue. This is in contrast to a study in South Africa where ART uptake for SC was high [22]. In the era of treat all and U = U, some might wonder why SC techniques matter since if viral suppression is achieved, the risk of HIV transmission to partners should be eliminated. However, what this research highlights is that simply providing ART will not ensure that all couples are achieving the full benefits of treatment when trying to get pregnant. This is supported by findings from a South African SC clinic where it was found that many clients enrolling for SC services were not virally suppressed [22]. While viral suppression may be the most sustainable and relevant long-term goal, participants expressed apathy about ART use, making SC approaches to compliment ART use even more applicable. In addition, even when only the woman living with HIV attends SC services, her engagement is still beneficial and can lead to ART initiation, improvements in treatment adherence, and education around other SC techniques that can help prevent partner transmission [22].

#### Strengths and limitations

These study findings have strengths and limitations. A strength of this research was that local researchers, research assistants, and WLHIV shaped this study so that local voices and perspectives guided study design and implementation. Prolonged engagement and collaboration with BONEPWA+, WLHIV, and the local team of researchers further improve the trustworthiness of these results. FGDs were conducted in predominantly urban and peri-urban areas. While those in rural settings might express different sentiments, almost 70% of the population in Botswana is urban, making the experiences of this group meaningful for offering future programs. However, these findings are not generalizable to all contexts. Also, all participants were recruited from PLHIV support groups. A strength of this approach is that WLHIV who were part of support groups likely felt comfortable discussing personal attitudes in the group setting. However, recruiting from support organizations may also have introduced potential biases. For example, support group members may be more empowered and more knowledgeable about SC options because of their group involvement. However, much of what was discussed was hypothetical as most of the SC methods were not available to women nor did they have prior experience with these methods. Therefore, what is reported here represents women’s initial reactions about various SC methods, which may or may not translate into use of a method after greater consideration or discussions with one’s partner. In addition, women who use support services may not be representative of all WLHIV. The experiences and attitudes of support group members may represent a “best case scenario” with regard to SC because they may feel more support around using SC methods, making their experiences less representative. There may be less interest or differences in preferences around SC methods among the general population. While the study design would have benefitted from the inclusion of men/sero-different couples, this study was conducted amongst WLHIV. While we recognize that most decisions about SC are made as a couple and that men have a dominant role in childbearing decisions in many sub-Saharan African contexts, there were concerns among community partners about potential disclosure challenges when trying to recruit sero-different partners. Also, mixed groups of men and women had the potential to prohibit women from freely speaking about SC and the challenges they face when trying to achieve their reproductive desires. As noted elsewhere, women access SRH services more frequently than men, making their perspectives especially important [22, 43–45]. Since some of the main concerns about SC focused on whether methods would be acceptable to partners, future studies among couples should be conducted to provide nuance about partner dynamics and SC. The study also did not include women not living with HIV who have male partners who are living with HIV. This would have been an interesting comparison group to the WLHIV and future studies should compare the SC preferences of these two types of women.

## Conclusion

The lack of SC counseling and services is a significant gap in the current system of care for PLHIV across much of sub-Saharan Africa and SC services are urgently needed by those who wish to conceive [40]. Despite the known benefits of SC methods, these techniques are not being offered as the standard of care in Botswana and many similar contexts. Dealing with fertility and childbearing should be a routine part of HIV care. We should strive to normalize childbearing for PLHIV and offer SC as part of a continuum of services that includes family planning counseling, contraceptive services, a range of SC options, and PMTCT. WLHIV in Botswana had limited knowledge about SC methods but were interested in these techniques. Education and couples SC counseling will be needed in order to scale-up SC method use. Future research must focus on effective strategies for engaging male partners in SC counseling, addressing threats to traditional masculinity, and examining the cultural acceptability of methods. Finally, offering couples a range of SC options so they can mix and match techniques and can weigh the challenges and benefits of each is key and can help couples engage in the least possible risk for the mother, her partner, and child.

## Supporting information

S1 File (DOCX)

S2 File (DOCX)

S3 File (DOCX)
