# Supplementary material for: “I did not know about all these”: Perceptions regarding safer conception methods by women living with HIV in Gaborone, Botswana
Source: PLoS One. 2020 Dec 1;15(12):e0242992. doi: 10.1371/journal.pone.0242992 (PMC7707558; doi:10.1371/journal.pone.0242992)
Supplement: S2 File — (DOCX) [file pone.0242992.s002.docx]

**Safer Conception Study: Focus Group Discussion Guide**

**I. Introduction**

***Facilitator introduction:***

Hello. My name is _______ and my name is _______ and we will be leading our group today. I am a ________ and I work at _______. We would first like to thank you for taking the time to talk with us today. Your thoughts and opinions are very valuable and we appreciate your willingness to help us in our efforts to understand issues around safer conception for PLHIV in Botswana. Our group will last about 1 hour today.

***Reasons for being here:***

Thank you very much for agreeing to participate in today’s focus group. We are here today to learn more about your thoughts and views about motherhood, pregnancy planning, stigma, and safer conception for PLHIV in Botswana.

***Ground rules: Confidentiality & Respect each other’s opinions***

Since all of you have different backgrounds and life experiences, you may not all agree with the information and ideas that are presented. This is okay and something that we expect. We do want to make sure that all participants feel safe in today’s focus group so we ask that you treat each other with respect and that you do not say anything to another participant that may insult or harm him/her in any way. We also want everyone to feel comfortable expressing their points of view, so we are asking you to not talk about anything that a particular person said in this group to others after the group is over. So basically what is said in the group should stay in the group. This will help to protect everyone’s confidentiality and to create a safe and informative group. Do you have any questions before we get started?”

***Participant introductions:***

We are going to begin today by going around the room and introducing ourselves to each other. In order to do this I would like you to share:

1. Your first name, or some other name that you would like us all to use when talking with you during our session today.
2. Your age
3. How many children you have

Please remember that all participant information will be kept completely confidential. Any names used will be deleted from the transcripts and notes.

**II. Importance of pregnancy/ motherhood**

*Goal: Explore the role of pregnancy/ parenthood in identity formation for PLHIV in Botswana.*

1. In general, how important/not important is parenthood in Botswana?

*Probes:*

How does pregnancy/parenthood affect womanhood/manhood or your standing in the community? Can you give me an example of seeing this in your community?

- Do women feel pressure to have children? From whom (individual, partner, family, etc)?
- How do you/people feel about people who don’t have children? Is it looked down upon if women don’t have children?
- How important or unimportant is having a child in new intimate relationships? Why?

2. What effect does being a PLHIV have on the importance of being a parent in Botswana?

*Probes:*

- How does HIV affect the need/expectation for children? At the personal level? In relationship? Families? Community? Culturally?
- Can you give me an example of messages you have heard in your community/ from healthcare providers about WLHIV having children?

**III. Salience of pregnancy planning**

*Goal: Explore the phenomenon of planned pregnancies and what this construct means for PLHIV in Botswana.*

3. I would like to learn more about if WLHIV/PLHIV in Botswana plan for pregnancy. What would it mean for a woman to plan her pregnancy?

*Probes:*

- Please tell me, if/how do you think most WLHIV/PLHIV in Botswana plan their pregnancy?
- Who plans for pregnancy? (men, women, as an individual, couple)

**IV. Information about Safer Conception options/methods and Motivation to use Safer Conception options/methods**

*Goal: Examine the types of information about SC that PLHIV need in order to use/ consider using SC. Examine the motivation to use SC (the positive and negative personal, interpersonal, and societal consequences of having children/ using SC).*

5. There are various methods/strategies that WLHIV/couples can use when they want to get pregnant and one or both partners are living with HIV. These strategies, known as safer conception, can help reduce the chance that a sexual partner will become infected with HIV when a couple has unprotected sex while trying to get pregnant.

**Activity:** Present SC cards with brief description of each approach. Break group up into smaller groups (perhaps 2 people). Pass out a set of cards to everyone.

In your group, please take a look at each of these cards. Each card briefly describes a safer conception method. As you review each card, please think about which methods interest you, which ones you would want more information about, and what additional information PLHIV would need to be able to use these methods. We invite you to discuss your ideas with your partner. After you have reviewed each method, put the cards in a pile based on which method you would be most willing to use – with the one you like best on the top, and the one you like least on the bottom. If there are any you definitely would not use, put those in a separate pile.

Group leader - When the group comes back together, gather reactions to each SC method.

Now that you have had some time to look at these methods, please tell me, what do you think of these methods?

*Information Probes:*

- Have you heard of any of these methods?
- Which methods would you want more information about? Why?
- What information would PLHIV need/want to come for these methods (pregnancy in general? HIV transmission? Specific methods and how they work? The effectiveness of those methods? Their reproductive rights to have children)?

Now let’s look at the order you put the cards in.

*Motivation Probes:*

- You picked X method as your top choice – why? What is good about it? Do others agree/disagree?
- You picked X method as your last choice, why? What is bad about it? What would need to change to make X method your top choice? Do others agree/ disagree?
- Which method do you think your partner would be willing to use? Why?

6. I want to talk about some of the positive and negative things that might happen if you used one of these SC methods. *(pick specific methods to discuss)*

*Probes:*

What good things might happen as a result of using _________ SC method?

What bad things might happen as a result of using ___________ SC method?

**V. Behavioral skills needed to use Safer Conception methods**

*Goal: Examine the behavioral skills needed to use SC methods.*

7. **Activity continued**: Lets look at the back of the cards. Each method has some skills listed that you might need in order to use that method. I would like to go through each SC method and think about whether you think these are the right skills needed to use that method.

*Probes:*

- Do you agree with the skills listed?
- What additional skills should be added? Taken away?
- Are the skills that are listed do-able?

**VI. Interest in Safer Conception services**

*Goal: Explore whether there is interest in SC services, preferences for how these services should be offered, and barrier and facilitators to uptake.*

8. When some people are considering becoming pregnant, they can go to speak with a healthcare worker and receive something called pre-conception or safer conception counseling. This is where a healthcare worker looks at your health status and discusses options for reducing the risks of HIV tranmission to your partner and infant. Please tell me, how interested/not interested you would be in using safer conception services that could protect your partner from HIV infection when you are trying to get pregnant.

*Probes:*

- If SC options were available in clinics, how likely would you be to use them?

What would be your level of comfort in going to a HC to seek those services before becoming pregnant?

9. If we were to create services at health centers in Botswana to support SC, what services would you want or need?

*Probes:*

What would the ideal SC services look like or include?

Where should SC services be offered?

How should these services be offered? Is it better for services to be integrated in regular care or separate?

- Who should offer these services? (for example - staffed by doctors, nurses, others?)
- What would be the most important thing in creating these services?
- How can we reach women when they are not yet pregnant, but they might be considering a pregnancy?
- When should these services be offered?

10. If SC methods became available at a clinic near you, what kind of things would help you/ make it easier to use SC methods? What kind of things would make it harder to use SC services? What things keep people from using SC services?

*Probes:*

- Partner involvement? Navigating health services? Frequent reminders from HCWs?
- Availability – hours / location
- Access
- Partner involvement low

Can you describe any rumors that would prevent people from coming for these services?

**VII. Closing question**

*Goal: To end the focus group on a positive note.*

11. You have provided us with a lot of very important information today. Our last request is to tell us more about your experience of being a mother/father/ parent. Please tell us what you like about being a parent?

### **VIII. Conclusion of Focus Group**

Thank you again for this very helpful information. Before we go today I would like to give everyone the opportunity to share with me any other thoughts you have about being a parent, pregnancy planning, stigma, or safer conception.

Do any of you have anything else to add that would help us to understand these topics better?
