# Supplementary material for: “I did not know about all these”: Perceptions regarding safer conception methods by women living with HIV in Gaborone, Botswana
Source: PLoS One. 2020 Dec 1;15(12):e0242992. doi: 10.1371/journal.pone.0242992 (PMC7707558; doi:10.1371/journal.pone.0242992)
Supplement: S3 File — (DOCX) [file pone.0242992.s003.docx]

**Patlisiso ka boimana jo bosireletsegileng: Bokaedi ja puisanyo ya setlhopha**

**I. Matseno**

*Kitsiso ya moeteledipele:*

Dumelang. Leina la me ke _______,nna leina la me ke _______,re tla bo re eteletse pele puisanyo ya setlhopha gompieno. Ke bereka ke le ________, ke berekela kwa _______. Re tla rata pele go le lebogela go tsaya nako go tla go bua le rona. Dikakanyo le megopolo ya lona e botlhokwa thata e bile re lebogela ketleetsego ya lona thata go tla go re thusa go tlhaloganya dikgang tse di teng mabapi le boimana jo bo sireletsegileng mo bathong ba ba tshelang ka mogare wa HIV mo Botswana.Setlhopha sa rona se tla tsaya oura e le nngwe gompieno.

*Mabaka a gore re bo re le fa:*

Re a leboga go bo o dumalane go tsenelela puisanyo ya setlhopha gompieno. Re fa gompieno go ithuta ka dikakanyo tsa gago le megopolo ya gago ka go nna mmangwana,go baakanyetsa boimana, kgethololo, le boimana jo bosireletsegileng mo bathong ba ba tshelang ka mogare wa HIV mo Botswana.

*Melawana: Go somarela sephiri le megopolo ya ba bangwe.*

Ka lotlhe fa, lena le tlholego le dithuto tse di farologanyeng le maitemogelo a botshelo a a farologanyeng, ga le kake la dumalana lotlhe ka dikitso le dikakanyo tse le tla di bewang pele. Seo ke se re se solofetseng, ga go molato. Re ya go netefatsa gore mongwe le mongwe o ikutlwa a babalesegile fa, ka jalo re kopa gore le tlotlane, go seka ga nna le ope yo o tla buwang sepe se se tla amang kana se utlwisa yo mongwe botlhoko. Re batla gape gore mongwe le mongwe a gololesege go ntsha maikutlo le megopolo ya bone, ka jalo re kopa gore ope a seka a bua ka se yo mongwe a se buileng mo setlhopheng morago ga puisanyo. Se se tla thusa go somarela sephiri samong ka rona le go nna le setlhopha se se babalesegileng, se fa kitso. A go na le mongwe yo o nang le potso pele re simolola?

*Kitsiso ya ba tsaya karolo*

Re tla simolola ka go ipolelelana mongwe le mongwe wa rona. Go dira se, ke tla kopa gore re ipolelane:

1. O na le nomoro ha pele ga ga go o ka e dirisa go ipolela, kana leina le o tla eletsang gore re le dirise mo puisanong ya rona gompieno.
2. dingwaga tsa gago
3. legore o na le bana ba le kae
4. O na le lebaka le lekae o itsile seemo sa gago sa go re o tshela ka mogare.

Tswee tswee gakologelwa gore dikitso tsa motsaya karolo e tla nna sephiri. Maina otlhe a tla phimolwa mo mekwalong yotlhe morago ga puisanyo.

**II. Bomosola ja go ima /go nna mmangwana**

*Maikaelelo:Go sekaseka seabe sa boimana kana botsadi bo go bopengboleng jwa mo mothong yo o tshelang ka mogare wa HIV mo Botswana.*

1. Dikakanyo tsa gago ka kakaretso ke eng fela ka mosola wa go nna motsadi mo Botswana?

*Ditlhotlhomiso:*

Go ima kana go nna motsadi , go ama bosadi/ bonna jwa ga go mo morafheng? Ke kopa o mphe sekai ka se o se boneg mo morafheng wa ga go?

A bo mme ba na le go utlwa kgatello epe go tshola? Go tswa ko go mang (a le mongwe, mo kapelo, ba lelwapa, etc.)?

- Batho ba ikutlwa jang ka batho ba ba se nang bana? A ke sengwe se se tseelwang kwa tlase fa mme a sena bana?
- Go botlhokwa go le kahe go tshola mo kamanong e ntshwa le mokapelo? Mabaka?

2. A go na le kamano epe hela go nna motho yo o tshelang ka mogare le botlhokwa jwa go nna motsadi mo Botswana?

*Ditlhotlhomiso:*

Mogare wa HIV o amana jang le letlhoko/ keletso ya bana? Mo mothong ka bo ene? Mo kamanong? Mo go ba lelwapa? Mo morafheng? Ka setso?

Ke kopa go re o mphe sekai sa molaetsa o o utlwileng mo morafheng/ mo go ba botsogo ka WLHIV bat shola?

**III. Go tlhatlhoba boleng jwa go ipaakanyetsa boimana**

*Maikaelelo: Go tlhatlhoba go bona gore a go tshola ga batho ba ba tshelang ka mogare wa HIV go a kgethololwa le gore a go kgethololwa go na le seabe ka fa batho ba ba tshelang ka mogare wa HIV batsayang dikgang tsa tsholo ka teng.*

3.Ke ta rata go itse gore batho mo Botswana ba ba tshelang ka mogare ba ipaakanyetsa jang boimana?

*Ditlhotlhomiso:*

- Ka tswee tswee mpolelle, fa e le go re bontsi jwa bomme ba ba tshelang ka mogare? Batho ba ba tshelang ka mogare ba ipaakanyetsa boimana le gone jang?
- Ke mang o baakanyetsang boimana? (banna, basadi, motho k abo nosi, mme le rre)

**IV. Dikitso ka metlhale ya katoloso tsholo e babalesegileng le kgothatso ya go dirisa metlhale ya katoloso tsholo.**

*Maikaelelo: Go tlhatlhoba metlhale ya boimana jo bo babalesegileng le batho ba ba tshelang ka mogare ba e tlhokang go dirisa/ go akanyetsa go dirisa SC. Go tlhatlhoba dokgothatso tsa go dirisa SC (bomosola le bosula jwa motho o le nosi, ma gareng ga batho, le ditlamorago mo morafheng tsa go tshola o dirisa metlhale ya boimana jo bo babalesegileng).*

5. Go na le metlhale/ ditsela tse botsalano jwa WLHIV ba ka e dirisang fa ba batla go ima gape a le mongwe kgotsa botlhe ba tshela ka mogare wa HIV. Ditsela tse, di itsege jaaka katoloso tsholo e e babalesegileng, di ka thusa go fokotsa kgonagalo ya go fitisa mogare fa ditsala di nna le tlhakanelo dikobo fa ba leka go dira ngwana.

**Tiro:** Supa dikarata tsa metlhale ya boimana jo bo babalesegileng ka tlhaloso e e khutshwane ka motlhale mongwe le mongwe. Kgaoganya batho ban ne mo ditlhopheng tse di nnyane (batho ba le babedi). Aba dikarata.

Mo setlhopheng sa ga go, tswee tswee lebelela karata nngwe le nngwe. Karata ngwee le nngwe e tlhalosa ka bokhutshwane motlhale wa katoloso tsholo e e sirelesegileng. Fa o bala karata nngwe le nngwe, tswee tswee akanya ka metlhale e e go kgatlhileng, ke dife tse o ka batlang kitso e e feteletseng, le go re ke dikitso dife tse batho ba ba tshelang ka mogare a ka ditlhokang go kgona go dirisa metlhale e. Re go laletsa go bua ka dikakanyo tsa ga go le mokapelo wa ga go. Fa o fetsa go lebelela motlhale mongwe le mongwe, tlhatlaganya dikarata ka motlhale o o ka o eletsang go o dirisa go gaisa – motlhale o o o eletsang go fitisa o le kwa godimo, le o o sa eletseng go o dirisa o le kwa tlase. Fa go na le tse o ka se eletseng go di dirisa gotlhelele, di beele ka fa thoko.

Moeteledipele wa setlhopha: Fa setlhopha se bowa, kgobokanya megopolo ka metlhale ya SC.

Jaanong ka go re le nnile le nako ya go lebelela metlhale e, tswee tswee mpolelle dikakanyo tsa lona ka metlhale e?

*Ditlhotlhomiso tsa kitso*:

- A o kile wa utlwalla epe ya metlhale e?
- Ke metlhale efe e o ka batlang kitso e e feteletseng ka yone? Mabaka?
- Ke dikitso dife tse di PLHIV b aka ditlhokang/ eletsang go tlele metlhale (go ima ka kakaretso? Kanamo ya HIV? Metlhale e e lebaganeng le go re e bereka jang? Boleng jwa metlhale ee? Dithata tsa bone tsa go nna le bana)?

Jaanong a re lebagane le tsamaiso ya dikarata tsa ga go.

*Tlhotlhomiso ya kgothatso*:

- O tlhophile motlhale wa X e le o o eletsang go fitisa, lebaka? Ke eng se se molemo ka sone? A ba bangwe ba a dumelana/ ganetsa?
- O tlhophile motlhale wa X e le o o sa o eletseng go fitisa, lebaka? Ke eng se se bosula ka one? Ke eng se se ka fetolwang ka X go re e nne motlhale o o o eletsang go fitisa? A ba bangwe ba dumelana/ ganetsa?
- Ke motlhale ofe o mokapelo wag a go a ka lekang go o dirisa? Lebaka?

6. Ke batla go bua le lona ka dingwe tse di molemo le tse di bosula tse di ka diragalang fa o dirisa dingwe tsa metlhale ya SC e (*tlhopha metlhale e e lebaganeng*)

*Ditlhotlhomiso:*

- Ke dife tse di molemo tse di ka diragalang fa o dirisa ____________motlhale wa SC?
- Ke dife tse di bosula tse di ka diragalang fa o dirisa _____________motlhale wa SC?

**V. Dikitso tsa mekgwa tse di tlhokafalang go dirisa metlhale ya boimana jo bo babalesegileng**

*Maikaelelo: Go tlhatlhoba dikitso tsa mekgwa e e tlhokafalang go dirisa metlhale ya SC.*

7.**Tswelediso ya tiro:**A relebelleng kwa morago ga dikarata. Motlhale mongwe le mongwe o na le dikitso dingnwe tse o ka tswang o ka di tlhoka go dirisa motlhale yo. Ke ka rata fa re ka bua ka motlhale mongwe le mongwe wa SC, re akanye fa dikitso tse di ka tswa e le tsone tota tse di tlhokafalang go dirisa motlhale o.

*Ditlhotlhomiso:*

A o dumalana le dikitso tse di kwadilweng?

Go na dikitso tse dingwe tse di tlhokahalang? Tse di thswanetseng go ntshiwa?

A dikitso tse di kwadilweng di kgona go dirafatswa?

**VI. Kgatlhego ya dithuso tsa katoloso tsholo e e sireletsegileng**

*Maikaelelo: Go tlhotlhomisa fa go ka tswa go na le kgatlhego mo dithusong tsa SC, maikgethelo a go re dithuso tse di fiwa jang, dikgoreletsi le bathusi b aba ka firwang.*

8. Batho ba bangwe fa ba akanya ka go ima, ba ya go bona mongwe wa bongaka go gakololwa ka tse a tshwanetseng go di solofelwa pele ga boimana kana ka boimana jo bo babalesegileng. E ke yone nako e modiri wa botsogo a seka sekang botsogo ja gago, a bo a go gakolola ka ditsela tsa go fokotsa go abelana mogare wa HIV kwa mokapelong wa gago kana kwa leseeng. Wena o akanya eng ka go batla dikgakololo kwa go ba botsogo pele o ima?

*Ditlhotlhomiso:*

- Fa metlhale ya SC e ne e le teng mo dikokelwaneng, go na le kgonagalo e le kahe go re o e dirise?
- O ka ikutlwa o gololesegile go le kahe go ya go bona ba botsogo go kopa dithuso tseo pele fa o ka ima?

9. Fa re ka simolola dithuso mo dikokelwaneng mo Botswana go tshegetsa SC, ke dife dithuso tse o ka di batlang kgotsa wa ditlhokang?

*Ditlhotlhomiso:*

Dithuso tse di lebaganeng tsa SC di ka tswa di lebega jang kgotsa di akaretsa eng?

Dithuso tsa SC di fiwe kae?

Dithuse tse di fiwe batho jang? A go botoka dithuso tse di tsamaisiwe le dithuso tsa tsatsi le tsatsi tsa mo kokelwaneng kgotsa di kgaogannwe?

Ke mangy o a tshwanetseng go fa dithuso tse? (kaedi- dingaka, baoki, ba bangwe?)

Ke eng se se botlhokwa go fitisa ka go simolola dithuso tse?

Re ka ikgolaganya jang le bo mme ba ise ba ime, mme ban a le dikakanyo tsa go ima?

Dithuso tse di fiwe batho leng?

10. Fa metlhale ya SC e ka nna teng mo kokelwaneng e e gaofi le wena, go na le dilo dife tse di ka go thusang/ nolofatsang tiriso ya metlhale ya dithuso tsa SC? Go na le dilo dife tse di itsang batho go dirisa dithuso tsa SC?

*Ditlhotlhomiso:*

Go tsaya karolo ga mokapelo? A tsomana le dithuso tsa botsogo? Dikgakololo tsa mmereki wa botsogo kgapetse kgapetsa?

Nako e e teng?

Kgonagalo

Go tsaya karolo ga mokapelo mo go kwa tlase

A o ka tlholosa dikgang dipe tse di ka kgoreletsang batho go tla go kopa dithuso tse?

**VII. Potso ya bofelo**

*Maikaeleo: Go tswala puisanyo ya setlhopha ka thekegelo.*

11. O re file dikitso tse di botlhokwa thata gompieno. Kopo ya rona ya bofelo ke gore o re bolelele ka maitemogelo a gago a go nna mme/rre/ motsadi. Tswee tswee, re bolelele gore o rata eng ka go nna motsadi?

### VIII. Konelo ya setlhopha

Re leboga thata go re fa dikitso tse di botlhokwa tse. Pele re ka tsamaya, ke kopa go fa mongwe le mongwe tshono ya goka kgaogana le nna dikakanyo dipe fela ka go nna motsadi, go baakanyetsa boimana, kgethololo le go ima mo go babalesegileng.

A o na le sengwe se o batlang go se tlatsa go re thusa go thaloganya ditlhogo tse botoka? Fa go na le motlhale mongwe o o rateng o ka botsa mo go rona.
