# Supplementary material for: “I did not know about all these”: Perceptions regarding safer conception methods by women living with HIV in Gaborone, Botswana
Source: PLoS One. 2020 Dec 1;15(12):e0242992. doi: 10.1371/journal.pone.0242992 (PMC7707558; doi:10.1371/journal.pone.0242992)
Supplement: Supplementary file 3 [file pone.0242992.s001.docx]

Vaginal insemination during the fertile period

- Semen from an HIV-negative man is collected in a condom or cup
- Then, in a syringe, the semen is taken from the condom/cup and gently placed inside the woman
- Usually done at home.

Skills needed

- Start discussion with partner
- Engage partner in using method
- Partner disclosure
- Start discussion with healthcare worker
- Ability to plan for pregnancy
- Understanding of fertile days
- Ability to get to health center
- Engagement with HIV care services
- Confidence that you can use this method

Antiretroviral therapy (ARVs)

- Treating the HIV-positive partner with ARVs
- Checking their HIV viral load to make sure it is very low or ‘undetectable’
- Then the couple has sex without a condom during the days during the woman’s cycle when she is most likely to get pregnant

Skills needed

- Ability to plan for pregnancy
- Treatment adherence skills
- Engagement with HIV care to check viral load
- Starting discussion with healthcare provider
- Starting discussion with partner
- Understanding of fertile days
- Confidence that you can use this method
- Navigating access to health center for ARVs

PrEP (Pre-exposure prophylaxis)

- Treating the HIV-negative partner with ARV pills called pre-exposure prophylaxis, or PrEP, during the time they are trying to get pregnant
- Couple has sex without a condom during the days during a woman's cycle when she is most likely to get pregnant

Skills needed

- Ability to plan for pregnancy
- PrEP adherence for negative partner
- Access to PrEP services and PrEP care
- Partner disclosure
- Starting discussion with healthcare provider
- Understanding of fertile days
- Confidence that you can use this method

Timed unprotected intercourse

- Every month during a woman’s cycle, there are a few days when she is most fertile (most likely get pregnant).
- A woman can track her cycle to figure out the days when she is most likely to get pregnant.
- A couple then has sex without a condom during those days to try and increase the chance of getting pregnant.

Skills needed

- Ability to plan for pregnancy
- Understanding of fertile days
- Starting discussion with healthcare provider
- Starting discussion with partner/ negotiating condom use
- Confidence that you can use this method

Voluntary Medical male circumcision (VMMC)

- HIV-negative men have a surgical procedure where the foreskin of the penis is removed. This can reduce the chance of the man getting HIV during unprotected sex.
- Men choosing this method will need to wait at least 6 weeks after VMMC before having sex.

Skills needed

- Start discussion with partner
- Engage partner in using method
- Partner disclosure
- Ability to get to health center
- Confidence that you can use this method
